# Comparative Efficacy of Non-Invasive Therapies in Temporomandibular Joint Dysfunction: A Systematic Review

**DOI:** 10.7759/cureus.56713

**Published:** 2024-03-22

**Authors:** Hesham A Alowaimer, Sultan S Al Shutwi, Mohammed K Alsaegh, Ohood M Alruwaili, Abdullah R Alrashed, Salwa H AlQahtani, Mohammed S Batais

**Affiliations:** 1 Maxillofacial Surgery, Ministry of Health, Al-Qassim, SAU; 2 Dentistry, Qassim University, Al-Ras, SAU; 3 General Dentistry, Ministry of Health, Riyadh, SAU; 4 Dentistry, Imam Abdulrahman Bin Faisal University, Dammam, SAU; 5 General Dentistry, Saudi German Hospital, Jeddah, SAU; 6 Pediatric Dentistry, Alyamamah Hospital, Riyadh, SAU

**Keywords:** tmj pain, dental laser therapy, tmj, tmj surgery, tmj disorders

## Abstract

Temporomandibular disorder (TMD) is a multifaceted disorder impacting the temporomandibular joint (TMJ), causing substantial discomfort and functional limitations. This systematic review aims to comprehensively assess the effectiveness of non-invasive treatment modalities for TMJ dysfunction, prioritizing a definitive protocol to ensure patient safety and enhance quality of life. Employing the PRISMA guidelines, we meticulously analyzed 20 studies from a pool of 1,417 articles sourced from databases such as PubMed, Google Scholar, ScienceDirect, and Medline. These studies underscore the multifarious nature of TMD and the varied responses to treatments such as physical therapy, laser therapy, ultrasound and electrical stimulation, splint therapy, injections, and arthrocentesis. Notably, the review highlights the paramount importance of precise diagnosis, often through surface electromyography, followed by a tailored treatment approach integrating manual therapy, counseling, and splint therapy. The systematic analysis revealed that while certain treatments such as transcutaneous electrical nerve stimulation and low-level laser therapy showed limited efficacy, combination therapies, especially those involving manual therapy, counseling, and splint therapy, demonstrated substantial improvement in reducing pain, depression, and anxiety. The findings advocate for a non-invasive, patient-centric approach, emphasizing education and symptom management before considering more invasive procedures such as injections and arthrocentesis. The review identifies the need for more comprehensive, longitudinal studies to establish a standardized, evidence-based treatment protocol for TMJ dysfunction, aiming to improve patient outcomes holistically.

## Introduction and background

A crucial joint in the human body, the temporomandibular joint (TMJ) is classified as a diarthrodial joint that allows for extensive movement. Specifically, this joint involves the articulation between the mandibular condyle, part of the lower jaw, and the glenoid fossa, located on the temporal bone. A fibrous disc acts as a partition between these structures and creates superior and inferior joint cavities, which are lubricated by synovial fluid. The articular capsule, complemented by ligaments and muscles, facilitates intricate functional movements that are smooth and multidirectional. This capability is vital for effective chewing and speaking, and disruptions to this functional harmony will significantly affect a patient's quality of life, underscoring the importance of the TMJ. Temporomandibular disorders (TMDs) are a group of degenerative musculoskeletal conditions characterized by morphological and functional deformities. These abnormalities are related to the positioning and/or structure of the intra-articular disc, as well as dysfunction in the associated musculature [1,2]. The stomatognathic system comprises various anatomical structures that collectively enable essential functions such as opening the mouth, swallowing, breathing, phonation, sucking, and performing diverse facial expressions. Along with the TMJ, which is pivotal for jaw movement, this system includes the jaw and mandible, various muscle tissues and tendons, dental arches, salivary glands, the hyoid bone along with its connecting muscles to the scapula and sternum, and the muscles of the neck [3-5]. The proper functioning of the TMJ and its associated structures is crucial for facilitating jaw movement and managing joint stress from routine actions such as chewing, swallowing, and speaking. Individuals with TMDs often experience a range of musculoskeletal issues in the joint, which can cause morphological and functional deformities [6]. TMD is characterized by abnormalities in the position or structure of the intra-articular disc and dysfunction in the related muscles. Those experiencing orofacial pain from TMD may exhibit various symptoms and signs, such as joint pain, joint sounds, restricted or unusual joint motion, and cranial and/or muscular pain [1,7].

A TMD is a musculoskeletal disorder characterized by pain and discomfort in the muscles and joints involved in chewing and jaw movement. Despite the existence of various sub-diagnoses, such as myofascial pain and TMJ inflammation, many practitioners still view TMD as a single disorder. TMD is a prevalent condition that most commonly affects individuals between 20 and 40 years old. This age-specific prevalence can be attributed to several factors, including the physical and psychological stresses common in this age group, which may contribute to the onset or exacerbation of TMD symptoms. Approximately one-third of the population experiences at least one TMD-related symptom, such as jaw pain or clicking. TMD is often perceived as a condition influenced by repetitive movements that affect the structures involved in chewing. As with other musculoskeletal disorders, TMD patients benefit from therapeutic approaches commonly used for similar conditions [8,9].

Clinicians continue to face challenges in diagnosing and effectively managing the primary cause of non-dental pain in the maxillofacial region, namely TMDs. Despite extensive clinical research efforts, TMD remains challenging to treat due to its nature as a comprehensive term encompassing various conditions with intricate origins. These conditions are further complicated by symptoms that vary widely in severity, adding to the diagnostic and therapeutic complexities [7]. Interestingly, while certain signs and symptoms of TMD may spontaneously resolve without intervention, others persist for extended periods, even after exhausting all available treatment options. Furthermore, while some TMD cases have a clear physical basis, many others involve significant biopsychosocial elements, including psychological symptoms such as depression and anxiety. Over time, a multitude of treatment modalities have been suggested, with some becoming obsolete and others gaining popularity. However, a single solution for all cases of TMD is not feasible due to the diverse array of symptoms associated with the disorder. The literature reveals controversies surrounding the diagnosis and management of TMD, and treatment choices are often heavily influenced by the healthcare provider's expertise and experience [10,11].

Non-surgical treatment is considered the best and most effective approach for managing TMDs in more than 80% of patients. There are numerous non-surgical treatment options available, and they often require the collaboration of a multidisciplinary team comprising multiple specialist practitioners. This cooperative approach ensures comprehensive care that addresses the various aspects of TMD.

Published reports indicate that approximately 5% of patients receiving treatment for TMDs eventually require surgery. Currently, there is a variety of surgical procedures available for TMD, ranging from less invasive options, such as TMD arthrocentesis and arthroscopy, to more complex procedures, such as arthrotomy, which involves open joint surgery. In line with the recommendations of oral and maxillofacial surgeons who specialize in this field, patients are typically advised to exhaust non-surgical treatment options before considering surgery [12].

The primary objective of this systematic review is to critically evaluate and compare the efficacy of various non-invasive therapies for managing TMJ dysfunction/arthralgia. The main goal of the research strategy is to effectively answer the question, "Which non-invasive treatment protocol is the most effective in addressing TMJ dysfunction/arthralgia?"

## Review

Methodology

 *Literature Search*

To gather relevant articles for our systematic review, we conducted searches on PubMed, Google Scholar, ScienceDirect, and Medline using the keywords “TMD” OR “TMJ Dysfunction” OR “Treatments” OR “Therapies” AND “Non-Invasive Therapy.” We adhered to PRISMA guidelines throughout the analysis to ensure precise reporting. Initially, we identified a total of 1,437 records. We applied filters to select English language publications, research articles, and clinical trials, resulting in the exclusion of book documents and review articles focused on non-invasive treatments for TMJ injuries, deformities, and dysfunctions. After applying these filters, 611 studies were screened for further relevance.

Upon a detailed examination of these studies, 229 articles were shortlisted for in-depth full-text evaluation. The remaining 442 articles were excluded for various reasons, including methodological shortcomings, poor quality or bias, inconsistency or irrelevance of outcomes, and being outdated or superseded by more recent research. After evaluating the 229 shortlisted studies, an additional 209 were excluded. Ultimately, only 20 studies met our specific criteria and were included in the review, proving to be most relevant to our study design and research objectives.

**Figure 1:**
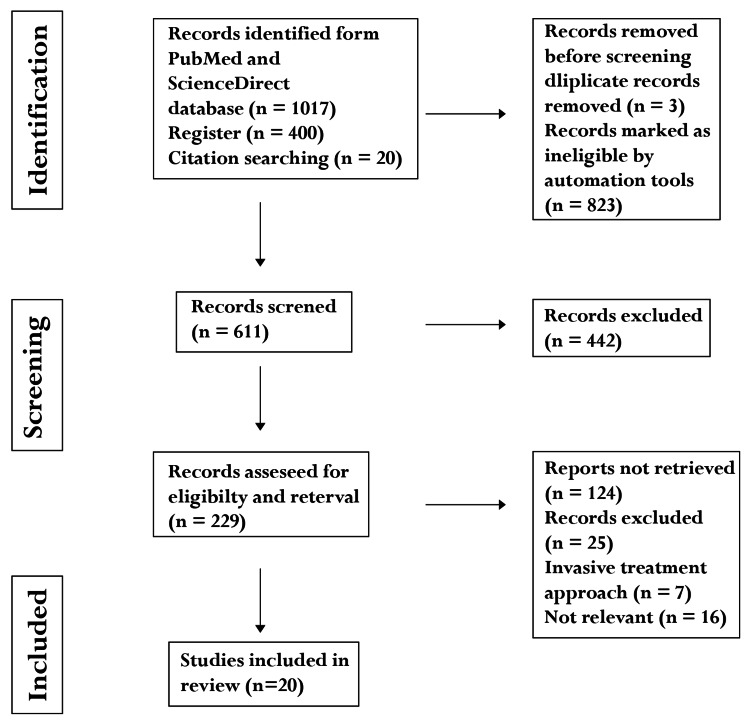
PRISMA flowchart PRISMA, Preferred Reporting Items for Systematic Reviews and Meta-Analyses

Figure 1 presents a flowchart illustrating the search methodology.

To assess the efficacy of various non-invasive and minimally invasive treatments, the researchers used mandibular muscle mobility, maximum pain-free mouth opening, and pain levels as outcome measures. A total of 17 articles were removed from the selected studies based on missing information about the outcomes of interest. Next, p-values that were obtained from paired t-tests and/or ANOVA were considered for analysis. However, in some studies, other factors were also considered to compare the effectiveness of different treatment methods [13-19] (Table 1).

**Table 1:** Characteristics of in vivo studies on humans investigating the efficacy of non-invasive treatments in TMD patients HA, hyaluronic acid; MMO, maximum mouth opening; NA, not reported; TMD, temporomandibular disorders; TMJ, temporomandibular joint; VAS, visual analog scale

First Author, Year	Type of Study	Baseline Characteristics (Gender, Age, Ethnic Group)	Non-Invasive Treatment	Frequency of Treatment	Duration of Intervention	Description of Application	Outcome Measures	Conclusion
Donnell et al., 2015 [13]	Randomized, placebo-controlled, single-blind clinical trial	24 female individuals with TMD, aged between 18 and 65 years.	High-definition transcranial direct current stimulation was used. Pain and motor dysfunction can be triggered in the brain without surgery.	Each session was 20 min, 5 sessions/day for one week.	Treatment lasted for 1 week, and follow-up was done at 4 weeks.	High-definition transcranial direct current stimulation montage targeted the primary motor cortex for the head and face area. It stimulated the side opposite the worst TMD pain. Electrodes were secured with a cloth cap and conductivity gel.	VAS, McGill Pain Questionnaire, PainTrek, Positive and Negative Affect Schedule, and pain-free mouth opening.	The therapy group demonstrated a significant decrease of 50% or greater in VAS scores, improvements in pain-free mouth opening, and reductions in pain area and intensity on the contralateral side of stimulation.
Conti, 1997 [14]	Double-blind pilot clinical trial	20 patients were divided into myogenous and arthrogenous groups. The mean age was 39.85 years.	Low-level laser therapy	Laser treatment was performed once a week.	3 consecutive weeks	Weekly sessions were done for 3 weeks using an 830 nm Ga-Al-As laser. The laser treatment delivered 4 joules of energy per session and was applied for 40 seconds at 100 mW directly to the affected area. The control group received a placebo treatment.	VAS, total vertical opening, right lateral excursion, left lateral excursion, and protrusive excursion.	Myogenous pain patients experienced a notable reduction in pain, as evidenced by lower VAS scores. Arthrogenous pain patients saw improvements in total vertical opening and increased protrusive and left lateral excursions.
Ekici et al., 2022 [15]	Randomized double-blind, placebo-controlled clinical trial	70 individuals aged between 18 and 70 years of age with myogenic TMDs.	High-intensity laser therapy.	The therapy was administered 5 days a week for 3 weeks.	15 days, with each session lasting 15 minutes.	Patients were randomized into two groups: the high-intensity laser therapy group and the control group.	Pain intensity, jaw function, MMO, functional disability, and quality of life through the oral health impact profile.	Therapy resulted in increased MMO and decreased VAS in the treated group compared to the control group.
Al-Quisi et al., 2023 [16]	Randomized double-blind, placebo-controlled clinical trial	60 individuals with myogenic TMD, aged 19 to 22 years, comprising 50 females and 10 males.	Red light-emitting diode therapies vs. laser therapy	Patients were evaluated across four visits, with a one-week interval between each visit.	One-week intervals for a month.	Patients were grouped into a placebo group, a group subjected to red light-emitting diode therapies, and a group treated with low-level laser therapy.	For the red light-emitting diode, pain levels were assessed for 5 minutes at each tender point. For low-level laser therapy, the pressure was applied for 30 seconds on the affected points.	In red light-emitting diode therapies and laser therapy, compared to the placebo group, there was a significant decrease in both pain levels and trigger points.
Gębska et al., 2023 [17]	Randomized clinical controlled trial	186 female patients with TMD, aged between 20 and 45 years.	Magnetostimulation, magnetoledotherapy, magnetolaserotherapy, and various forms of manual therapy.	Daily treatments were administered over 10 days, excluding weekends.	Each treatment session lasted 12 minutes.	Treatments were categorized into seven groups: magnetostimulation, magnetoledotherapy, magnetolaserotherapy, and four types of manual therapy combined with or without self-therapy. These interventions aimed to evaluate their effect on the bioelectrical function of the masseter muscle in patients with TMJ pain and limited mobility.	MMO, minimal important differences, generalized estimating equations regression model, lateral movement, pain, and muscle activity.	The study showed significant improvements with minimal important difference values indicating enhanced jaw function and pain reduction. MMO improved by 1.46 mm, lateral movement by 0.60 mm, and pain intensity decreased. Muscle activity measures also demonstrated substantial improvements.
Zhang et al., 2020 [18]	Randomized clinical controlled trial	The treatment group comprised 10 males and 10 females with disc displacement without reduction and TMD, aged 25-38 years. The control group comprised healthy individuals matched by gender and age.	Transcutaneous electrical nerve stimulation	Participants performed four continuous repetitive jaw movements in five sequential sessions, with 3-second intervals between each session.	The transcutaneous electrical nerve stimulation application lasted for 45 minutes, with stimulation intensity set below the pain threshold.	Transcutaneous electrical nerve stimulation was administered to reduce jaw pain and improve function in TMJ disorder patients.	Primary outcome: change in the intensity of jaw pain over 30 days, rated on a 0-10 scale. Secondary outcomes: changes in psychological distress, assessed by MMO and global severity index scores, respectively.	Compared to other groups, the test group showed significant pain relief and improvements in opening range and movement velocity during repeated open-close and horizontal movements.
Santana-Penín et al., 2023 [19]	Placebo-controlled randomized clinical trial	77 participants were split into two groups: 39 received equilibration therapy and 38 were given a placebo, predominantly female. The median age was 29.5 years. The equilibration group had chronic orofacial pain and TMD.	Remodeling dental anatomy vs. sham therapy	Two sessions.	The first session was 90 minutes long, and the second one was 30 minutes long.	The application focused on removing premature occlusal contacts to balance occlusion and adjust the lateral guidance angle, enhancing chewing on the non-used side. This dental process aimed to redistribute occlusal forces, protect the TMJs, and minimize enamel removal. It involved an initial 90-minute adjustment and a follow-up session.	Jow pain score, MMO, and psychological distress.	Over 6 months, equilibration therapy markedly reduced the intensity of jaw pain in patients with chronic TMD. This treatment led to a significant decrease in the MMO score to 2.1, demonstrating a significant improvement in the patient’s ability to open their mouths without assistance.
Zhang et al., 2023 [20]	Clinical trial (prospective)	The study included 72 TMD patients.	Low-intensity pulsed ultrasound.	One session daily, for 15 minutes.	1 week.	72 TMD patients were divided into two groups: 36 with unilateral masticatory myositis (26 females, average age 32.2 years; 10 males, average age 35 years) and 36 with unilateral joint synovitis (28 females, average age 33.9 years; 8 males, average age 31.5 years). Low-intensity pulsed ultrasound was utilized for physical stimulation of TMJ muscle regions.	Fricton TMD index, VAS, pressure difference of precision manometer.	Significant reductions were observed in the Fricton index for both masticatory myositis and synovitis groups, with VAS scores and pressure differences between affected and healthy sides also showing significant decreases.
Marques et al., 2023 [21]	Double-blind randomized feasibility study	The study involved a treated group comprising 10 individuals with an average age of 35.62 years, and a placebo group comprising 11 individuals with an average age of 41.5 years. Both groups were diagnosed with TMD.	Photobiomodulation Auriculotherapy	One session per week	1 month.	The protocol used photobiomodulation therapy for TMD, with a 660 nm wavelength and 100 mW power, applied once weekly for four weeks. Each session targeted specific points for 40 seconds, aiming to reduce TMD symptoms.	Diagnostic criteria for TMDs.	There were significant improvements in the treated group, with a 75% reduction in pain intensity and significant post-intervention improvements in pain and ability to work.
Melo et al., 2020 [22]	Randomized clinical trial	The study involved 89 participants (72 females, 17 males) with an average age of 28, diagnosed with TMD.	The four types of treatment are occlusal splint, manual therapy, counselling, and occlusal splint plus counselling.	40-minute sessions conducted twice a week	1 month	Participants were randomly assigned to groups and given a new non-invasive treatment each week for one month. Before and after the intervention, the patient's levels of anxiety and pain were assessed using standardized instruments.	VAS, Hospital Anxiety and Depression Scale, the Beck Anxiety Inventory, and the State-Trait Anxiety Inventory.	Therapy was effective in significantly reducing both pain and anxiety in all four groups.
Cuccia et al., 2010 [23]	Randomized controlled trial	50 patients with TMD aged 18 to 50 years.	The first group underwent osteopathic manual therapy, and the second group underwent conventional conservative therapy.	The osteopathic manual therapy sessions were conducted for 15 to 25 minutes per session.	The frequency of these sessions was set at intervals of 2 weeks throughout the study period. The follow-up sessions were conducted at 6 months and 8 months.	The osteopathic manual therapy protocol for treating TMDs included gentle techniques such as myofascial release, balanced membranous tension, muscle energy, joint articulation, high-velocity low-amplitude thrust, and cranial-sacral therapy.	VAS, MMO, and temporomandibular index.	The patients in the osteopathic manual therapy group had significant reductions in medication use, decreased pain levels, and enhanced jaw function.
Huang et al., 2014 [24]	Clinical trial, a case series	20 patients, averaging 34 years of age.	Laser acupuncture	Once per week	The therapeutic course ended when the patients felt like they no longer required treatment or when the patients showed no symptom improvement after three treatment courses.	Diode K-laser was applied on four acupuncture points, including three standard ipsilateral local points and one contralateral distal point. Pain intensity was measured before and after the intervention.	VAS and MMO.	The therapy resulted in a complete to partial reduction of pain without any side effects. Pain reduction after the intervention was very significant.
Tuncer et al., 2013 [25]	Randomized controlled trial	40 adults, comprising 31 females and 9 males aged 18 to 72 years, with TMD.	Home physical therapy vs. manual therapy plus home physical therapy.	Exercises were done three times a week, with each session lasting 30 minutes.	One month	Patients were divided into two groups depending on the therapy method. For the manual therapy combined with the home physical therapy group, the treatment protocol included soft tissue mobilization, TMJ mobilization, TMJ stabilization, coordination exercises, cervical spine mobilization, and post-isometric relaxation and stretching techniques for the masticatory and neck muscles.	VAS and MMO.	The manual therapy plus home physical therapy group had a greater improvement compared to the home physical therapy only group, with a 59.2% reduction in resting pain and a 91.3% reduction in stress-induced pain. Pain-free mouth opening increased by 10.0 mm in the combined therapy group versus 4.4 mm in the home therapy group.
Polat et al., 2020 [26]	Clinical trial, a retrospective study	45 TMD patients were divided into three diagnostic categories: osteoarthritis, disc displacement with reduction, and disc displacement without reduction, with an overall mean age of 29.74 years.	Arthrocentesis.	Single session	Outcomes were conducted at 1 and 6 months postoperatively.	A single arthrocentesis procedure was used in all patients regardless of their specific TMD type. This minimally invasive procedure aims to wash out the joint, remove inflammatory mediators, and improve joint mobility by injecting and then draining fluid from the joint space.	VAS, measurements of mandibular motion.	Significant improvements were observed across all types of TMDs, with a particular emphasis on disc displacement without reduction.
Calis et al., 2019 [27]	Clinical trial, a prospective type	9 individuals, comprising 4 males and 5 females, with an average age of 33.67 years. All individuals had muscular TMD marked by masticatory muscle deformities and hyperactivity.	Botulinum toxin type A was administered to patients who did not respond to initial treatments.	Single session	Single dose	Botulinum toxin injections were administered according to electromyography guidelines, targeting the masticatory muscles with specified doses to reduce pain and improve mouth openness.	Bite force, VAS, MMO.	There was a significant reduction in pain among patients receiving Botulinum toxin A injections for TMD. However, there was no significant change in bite force and mouth openness.
Gawriołek et al., 2015 [28]	Controlled clinical trial, a prospective type	32 females with TMD suffering from muscle disorder, mean age of 23.3 ± 4.8 years.	The treatment consisted of myorelaxation exercises and the use of a sublingual relaxation splint.	The treatment began with daily myorelaxation exercises. In the second stage, patients used a sublingual relaxation splint nightly while continuing the myorelaxation exercises.	Stage 1 involved daily myorelaxation exercises for 3 weeks. Stage 2 included follow-up assessments at baseline, after a 4-week control period, and then at 3 weeks, 3 months, and 6 months of treatment.	The treatment involved myorelaxation therapy, which combined a sublingual relaxation splint for nocturnal use with daily stretching exercises to alleviate TMD.	Outcomes were assessed with a computerized mandibular scanner, capturing the range, velocity, and direction of jaw movements, including opening, closing, lateral, and protrusive motions.	The therapy led to a 19% increase in mandibular opening and a 36% improvement in lateral movement, with a slight, non-significant decrease in protrusive movement. Pain and impairment significantly decreased, while joint clicking remained unchanged.
Virender et al., 2023 [29]	Clinical study, a prospective type	68 adult individuals with TMD with a mean age of 53 ± 16 years,	Intra-articular injections of HA, platelet-rich plasma, or injectable platelet-rich fibrin into the joint.	3 consecutive times/week for HA, once a week for platelet-rich plasma or injectable platelet-rich fibrin.	The HA group underwent three weekly sessions, while the platelet-rich plasma and injectable platelet-rich fibrin groups had one session each. Follow-ups were conducted at 6 and 12 months.	MRI-guided navigation ensured accurate botulinum toxin A injections into the lateral pterygoid, masseter, and temporalis muscles, emphasizing safety and precision with real-time monitoring.	MMO and VAS.	All treatment groups showed significant improvements in pain relief and jaw opening. The HA group demonstrated a significant improvement in MMO, while pain reduction was significant across all treatments.
Rodrigues et al., 2019 [30]	Prospective randomized controlled trial, blinded	21 participants with TMD were split into two treatment groups, with an average age of 45.57 years.	The first group underwent low-power laser auriculotherapy. The second group had occlusal splints.	The first group participated in weekly sessions, while thesecond group had two to three sessions.	The first group received treatments over 8 weeks. The second group had their follow-up visits scheduled for 48 hours and one week after treatment initiation.	Patients were divided into two groups: the low-power laser auriculotherapy test group and the occlusal splints control group. They were subjected to the respective treatments.	VAS, the graded chronic pain scale, and depression levels	Both treatments showed significant improvements in physical and emotional symptoms for TMD, with no significant difference in their effectiveness.
Pons et al., 2019 [31]	Controlled clinical trial, a prospective study	6 patients (5 females and 1 male), with an average age of 28.5 years, were diagnosed with persistent myogenic TMD.	MR-guided intramuscular injections of botulinum toxin A.	Single dose of botulinum toxin A.	One session with follow-up after 1 and 3 months.	Each lateral pterygoid muscle received an injection of 20 UI of botulinum toxin A, with the target being the center of the upper head. Additionally, 30 UI were administered in each masseter and 20 UI in each temporalis muscle.	Pain intensity, joint sounds, maximum interincisal opening.	Significant improvements in pain reduction and jaw opening in patients receiving MR-guided botulinum toxin injections for TMD, with a significant decrease in pain intensity and enhanced jaw functionality.
Martenot et al., 2019 [32]	Retrospective study	34 patients, including 31 females and 3 males, with a mean age of 35 years, for the treatment of persistent myogenic TMD.	Injection of botulinum toxin type A using intraoperative real-time navigation.	34 patients received a total of 51 injections, with 8 patients undergoing multiple treatments.	4-month intervals between each Botulinum toxin type A.	Botulinum toxin type A was injected into the lateral pterygoid muscle with 25 units per side. The masseter and temporalis muscles also received targeted injections based on clinical guidance.	Maximal interincisal mouth opening, joint sounds, and pain are assessed by a numerical rating scale.	Significant pain intensity decreased by about 65% at 1 month post-treatment, and joint sounds significantly decreased to 9.7% by 3 months. Additionally, mouth opening increased significantly. At 3 months, 63% of patients reported complete improvement.
BA et al., 2021 [33]	Controlled clinical trial	160 TMD patients aged 18 to 68 years.	Ultrasound therapy	Once-daily sessions for 5 days a week.	2 consecutive weeks. The follow-up period for assessing the outcomes of this treatment was conducted at 4 weeks and 6 months after the therapy sessions.	The ultrasound treatment group comprised 43 males and 37 females, and the control group consisted of 42 males and 38 females. For the treatment group, each treatment session featured three 5-minute ultrasound exposures, separated by 2-minute intervals between blasts.	Pain-free interincisal distance, mandibular movement, jaw noise, disability index, and the craniomandibular index.	Therapy significantly reduced pain and improved jaw function and the craniomandibular index in patients with TMDs. These benefits were observed both 4 weeks and 6 months post-treatment, with a low recurrence rate of symptoms.

In our systematic search and review process, roles were clearly defined to ensure efficiency and thoroughness. The first and last authors served as the main leaders and decision-makers. They oversaw the overall search strategy, aligning it with the study's objectives and resolving any arising conflicts or disagreements. Their role was crucial in maintaining the integrity and direction of the research. The other authors, each with their specialized expertise, conducted individual literature searches relevant to our study's scope. This collaborative effort allowed for a comprehensive and diverse data collection, ensuring thorough exploration of various topic aspects. In cases of conflicting data or differing opinions, the first and last authors would discuss these issues to reach a consensus, providing a balanced and well-considered resolution. This structured approach, with clear leadership and collaborative participation from all authors, ensured a systematic, unbiased, and comprehensive review process. This significantly contributed to the quality and reliability of our systematic review. Moreover, there are no conflicts of interest among the authors, and the research was conducted without any bias.

Analysis

Several factors were used to evaluate the effectiveness of non-invasive or barely invasive treatments in improving the symptoms of TMD: p-value, percentage of positive responses from patients, and the number of symptoms improved by a method. Different treatment methods were compared, and the effectiveness of combination therapies was also evaluated (Table 2).

**Table 2:** Efficacy of different treatments HA, hyaluronic acid; TMD, temporomandibular disorders; TMJ, temporomandibular joint

Reference	Treatment Method	Number of Applicants	Outcome Measures						Response in Patients	Side Effect
			Pain	MMO	Muscle Mobility	Depression	Anxiety	Joint Sounds		
Donnell et al., 2015 [13]	Non-invasive brain modulation of pain and motor dysfunction	24 patients	p = 0.04	p < 0.01	-	-	-	-	Therapy was significant improvement in the patients.	Side effects were mild, such as headache, neck and scalp pain, tingling, and a feeling of scalp burn. No severe side effects or skin lesions occurred. Generally, side effects were rare and minor.
Conti, 1997 [14]	Low-level laser therapy	20 patients	p < 0.02	p	p	-	-	-	Significant pain reduction in myogenous patients and improved jaw function in arthrogenous patients with laser therapy	The study did not report any adverse effects or complications
Ekici et al., 2022 [15]	High-intensity laser therapy	70 patients	p < 0.001	p < 0.001	p < 0.001	-	-	-	Therapy was effective in 47 % of patients.	The study did not report any adverse effects or complications
Al-Quisi et al., 2023 [16]	Light therapies vs. laser therapy	60 patients	p = 0.05	-	-	-	-	-	Both therapies were equally effective in reducing pain levels.	The study did not report any adverse effects or complications
Gębska et al., 2023 [17]	Surface electromyography and physical therapy	186 patients	p < 0.001	p < 0.001	-	-	-	-	Therapy was effective in 99% of the targeted cases.	The study did not report any adverse effects or complications.
Zhang et al., 2020 [18]	Transcutaneous electrical nerve stimulation	20 patients	p = .03	p = 0.033	-	-	-	-	Therapy was found to be significant in improving symptoms of TMD	The study did not report any adverse effects or complications.
Santana-Penín et al., 2023 [19]	Remodeling dental anatomy vs. sham therapy	77 patients	p = 0.004	p = 0.02	-	-	-	-	Remodeling dental anatomy was adequate in most of the cases.	No significant adverse events were noted. Nonetheless, hypersensitivity was observed in three individuals from the test group.
Zhang et al., 2023 [20]	Low-intensity pulsed ultrasound	72 patients	p < 0.001	-	-	-	-	-	Therapy was found effective in most of the treated cases.	The study did not report any adverse effects or complications
Marques et al., 2023 [21]	Photobiomodulation auriculotherapy	21 patients	p = 0.005	-	-	p = 0.02	p = 0.02	-	Therapy was found to be significant in improving symptoms of TMD.	Mild side effects included temporary discomfort at the treatment site.
Melo et al., 2020 [22]	An occlusal splint, manual therapy, counseling, the combination of an occlusal splint and counselling	89 patients	p < 0.001	-	-	p < 0.001	-	-	All therapies were effective in managing TMD symptoms.	The study did not report any adverse effects or complications.
Cuccia et al., 2010 [23]	Osteopathic manual therapy vs. conventional conservative therapy	50 patients	p < 0.0001	p < 0.0001	p = 0.046.	-	-	-	Osteopathic Manual Therapy was more efficient compared to conventional conservative therapy	The study did not report any adverse effects or complications.
Huang et al., 2014 [24]	laser acupuncture	20 patients	p = 0.0003	p < 0.001	-	-	-	-	Therapy was found effective in 85% of patients.	The study did not report any adverse effects or complications.
Tuncer et al., 2013 [25]	Home physical therapy vs. manual therapy and home physical therapy vs manual therapy alone.	40 patients (9 males + 31 females)	p < 0.001	p = 0.009	-	-	-	-	Manual therapy and home physical therapy were effective in 91.3% of the treated patients.	The study did not report any adverse effects or complications.
Polat et al., 2020 [26]	Arthrocentesis therapy: osteoarthritis, disc displacement with reduction, disc displacement without reduction.	45 patients	-	p = 0.008	p = 0.002	-	-	p = 0.001	Significant improvements were observed across all types of TMDs.	The study did not report any adverse effects or complications.
Calis et al., 2019 [27]	Botulinum toxin A injections	9 patients	p < 0.005	Improved	Improved	-	-	-	Therapy was effective in managing most of the treated cases.	The study did not report any adverse effects or complications.
Gawriołek et al., 2015 [28]	Myorelaxation therapy	32 patients	p = 0.05	p = 0.05	p = 0.05	-	-	-	A combination of drug-physical therapy-occlusal splint was 36% effective in the treated patients.	Mucosal trauma, prosthetic retention issues, and calculus accumulation were noted, with three subjects experiencing short-term discomfort after initial relaxation exercises. All complications were self-resolving.
Virender et al., 2023 [29]	Intra-articular injections of HA, or platelet-rich plasma, or injectable platelet-rich fibrin	32 patients	p = 0.01	HA p = 0.01	-	-	-	-	All treatment groups showed significant improvements.	The study did not report any adverse effects or complications.
Rodrigues et al., 2019 [30]	Low-power laser auriculotherapy vs. occlusal splints	21 patients	Reduced	Improved	Improved	-	-	-	Both therapies were equally effective in improving symptoms of TMD.	The study did not report any adverse effects or complications.
Pons et al., 2019 [31]	MR-guided intramuscular injections of botulinum toxin A	6 Patients	p<0.01	p<0.01	-	-	-	Improved	Significant improvement in pain and joint sounds, with a notable increase in mouth opening among patients.	The study did not report any adverse effects or complications.
Martenot et al., 2019 [32]	Botulinum toxin A injections into the lateral pterygoid muscles, the masseter, and the temporalis muscles	34 patients	p < 0.001	p = 0.008	-	-	-	p = 0.01	63% of patients reporting complete improvement.	In 9.8% of patients, symptoms may include worsening pain, headaches, muscle contractions, weakness, and paralysis of the upper lip. All were resolved by the 1-month follow-up.
BA et al., 2021 [33]	Ultrasound therapy	Test group: 80 patients	p < 0.01	p < 0.01	-	-	-	-	Therapy effectively reduces pain and enhances TMJ functionality and mouth opening.	The study did not report any adverse effects or complications.

Results

In affected individuals, TMDs have been observed to decrease the overall quality of life. Even the most resilient patients are bothered by the distressing symptoms associated with this condition. These symptoms include persistent jaw pain, frequent headaches, and difficulties in eating and speaking, all of which can significantly impact daily life.

Given the significant challenges posed by TMDs, it is essential to adopt a treatment approach that is both cost-efficient and effective while also prioritizing patient safety. Surgical treatments are associated with various complications, making conservative and non-invasive therapies more appealing due to their inherent benefits. Consequently, these therapies have become the preferred first-line treatment for TMDs. The conservative and non-invasive approach is primarily characterized by its focus on minimizing risks and educating patients. Instead of immediately resorting to invasive procedures, this first line of therapy concentrates on empowering patients through education about their condition and providing them with tools to effectively manage their symptoms [13-33].

This systematic review analyzed 20 studies that employed a range of treatments, including physical therapy, laser therapy, ultrasound, electrical stimulation, splint therapy, injections, and arthrocentesis. Across these studies, a total of 798 patients underwent treatment using non-invasive and conservative therapies.

Physical Therapy

Physical therapy treatments for TMDs include manual techniques such as mobilizations, stretching, and manipulations of the TMJ and cervical spine. They also involve modalities that improve tissue health, exercise guidance (including self-stretching and mobility strategies), and patient education on relaxation techniques, postural instruction, and parafunctional awareness. Wright and North suggest that patients with cervicogenic headaches or forward head posture or those who have never received physical therapy for TMD should be encouraged to undergo physical therapy. Regular practice of postural exercises has been shown to improve the symptoms of TMD [34].

Functional and physiological activities involving mandibular movements, such as opening and closing the mouth, mastication, swallowing, and the occurrence of centric and eccentric occlusal contacts between the teeth of the mandible and maxilla, are crucial. These functional movements greatly affect the quality of life and are considered key oral health parameters. The movements of the bilateral TMJ, along with the presence of the articular disk, play a crucial role in ensuring proper mandibular guidance [35].

In a clinical trial involving 78 patients, Gawriołek et al. investigated the effectiveness of myorelaxation therapy in treating TMJ malfunctions [28]. The study analyzed clinical findings, measurements of mandibular movement, and reported functional impairment. The findings indicated that myorelaxation therapy significantly improved efficacy. Notably, the opening and closing velocity and the range of opening and lateral movement showed considerable improvement after six months of treatment [28]. The study by Gębska et al. aimed to evaluate the efficacy of surface electromyography testing and manual therapy treatments in enhancing the bioelectrical function of the masseter muscle in individuals with restricted TMJ mobility [17]. The study revealed that therapeutic effectiveness could be effectively assessed using surface electromyography testing. It also emphasized including manual therapy treatments in the initial non-invasive intervention. The results indicated that manual therapy was more effective for pain relief and muscle relaxation compared to physical therapy [17].

Tuncer et al. conducted a comparative analysis to assess the effectiveness of home physical therapy versus a combination of manual therapy with home physical therapy. Their findings indicated that the combination of manual therapy with home physical therapy was more effective than home physical therapy alone [25]. Osteopathic treatment, classified as a physical therapy intervention, employs fine manipulative techniques that are less invasive than other interventions. These techniques are individually tailored to the patient's tissue quality and are designed to either maintain or restore the circulation of body fluids. The concept of osteopathic treatment as a form of manual medicine was first introduced by Andrew Taylor Still in 1902. Cuccia et al. undertook a study to compare osteopathic manual therapy with conventional conservative therapy to critically assess their respective efficiencies. Their research concluded that osteopathic manual therapy was more effective compared to conventional conservative therapy [23].

Laser Therapy

The efficacy of light therapies is contingent on the absorption of photons at a cellular level. The use of LASER (light amplification by stimulated emission of radiation) technology can initiate photochemical reactions at the mitochondrial level, triggering changes in cell metabolism and protein synthesis. Additionally, low-level light therapy is believed to stimulate the formation of new blood vessels, as well as increase collagen production and fibroblast cell activity [36]. Laser therapy is beneficial in treating dentin hypersensitivity, soft tissue disorders, musculoskeletal pain, and bone regeneration. Both low-level and high-intensity laser therapy are extensively used to treat TMDs. A study investigating the efficiency of low-level laser therapy in patients with TMJ disorders treated 20 individuals experiencing pain with an 830 nm Ga-Al-As laser device that delivered an energy of 4 joules. However, the results indicated that the therapy was not significantly effective [14]. In contrast, Ekici et al. evaluated the efficacy of high-intensity laser therapy in treating patients with myogenic TMJ disorders; 76 patients were randomized into two groups: a control group and a test group. The test group patients received high-intensity laser therapy and experienced a significant (47%) reduction in pain scores compared to the placebo group [15].

Rancan et al. conducted a clinical trial with 17 patients using stainless steel needles for acupuncture. This treatment was administered weekly for a total of 10 sessions and achieved a significant reduction in both visual analog scale (VAS) scores and TMD symptoms, thus indicating the effectiveness of acupuncture as a treatment option [37]. Huang et al. evaluated the clinical effectiveness of laser acupuncture in treating TMDs. In their study, 20 patients were treated with a diode K-laser, which has a wavelength of 800 nm, once a week. The results demonstrated that 85% of the patients experienced varying degrees of pain relief [24].

Al-Quisi et al. compared the efficacy of light therapy and laser therapy in reducing pain among individuals with TMDs [16]. It is proposed that low-level light therapy can potentially stimulate the formation of new blood vessels, increase collagen production, and enhance fibroblast cell activity. These effects, in addition to raising tissue temperature, can enhance microcirculation in the irradiated tissue, thereby effectively removing a majority of inflammatory mediators. The key difference between these various light therapies lies in the specific wavelength and optical power used. These factors directly influence the amount of energy delivered and the depth of light penetration through tissue [38]. Both LED (light-emitting diode) and laser treatments are effective in providing therapeutic relief for the myogenous symptoms of TMDs [16,38].

Ultrasound and Electrical Stimulation

Ultrasound has been extensively researched as a potential treatment for temporomandibular joint osteoarthritis (TMJ-OA) and hypoxia-induced chondrocyte damage in TMDs. This interest stems from the fact that low-intensity ultrasound acts as a stimulator; it promotes neovascularization, facilitates the differentiation of mesenchymal stem cells, and aids in the local release of angiogenic factors. These effects then improve blood flow in ischemic tissues [39,40]. In a clinical trial conducted by Ba et al., a total of 168 patients with TMD were divided into two groups for the study. The test group received ultrasound treatment. The treatment protocol involved a single daily application for five days a week over two weeks, with each session consisting of three 5-minute blasts and a 2-minute interval between each blast. The effectiveness of ultrasound as a treatment for TMD was established in this study; only 2.63% of patients experienced a recurrence of symptoms after six months of therapy [40]. The output frequency range of therapeutic ultrasound typically falls between 20 and 60 kHz. This treatment increases the stretch of the extracapsular soft tissue by generating deep heat at the joints, effectively treating joint contracture. Additionally, it aids in the stretching of soft tissue by reducing the viscosity of collagen, thereby decreasing non-acute pain, muscle spasms, and tendonitis. It also facilitates the breakage of calcium deposits in bursitis and decreases the firing of type II muscle spindles [33]. The primary goals of using electrical stimulation devices for treating TMDs are to provide pain relief and address muscle hyperactivity or spasms. These devices utilize either transcutaneous electrical nerve stimulation (TENS) or high-voltage galvanic stimulation. TENS employs a low-voltage, low-amperage, biphasic current at varying frequencies, while high-voltage galvanic stimulation uses a higher voltage (>150 V), low-amperage, monophasic current at varying frequencies [41,42]. Zhang et al. explored the efficacy of low-intensity pulsed ultrasound (LIPUS) in treating synovitis and masticatory myositis in TMD. They found that the therapy was effective in most tested cases after one week of LIPUS treatment [20]. Zhang et al. also studied the impact of TENS on jaw movement-evoked pain in individuals with TMJ disc displacement. They observed a reduction in movement-evoked pain in TMJ patients with disc displacement without reduction (DDwoR), suggesting that TENS reduces activity-related pain in these patients [18]. Donnell et al. determined the impact of motor cortex high-definition transcranial direct current stimulation (HD-tDCS) on clinical pain and motor measures in TMD patients. Twenty-four females underwent five daily, 20-minute sessions of active 2 milliamps HD-tDCS. Compared to the placebo group, the HD-tDCS group reported significant improvement in motor measurements and clinical pain [13].

Splint Therapy

One of the primary goals of splint therapy is to restore the vertical dimension of occlusion, which involves properly aligning the teeth and jaw. Occlusal splints are removable artificial occlusal surfaces used for diagnosis or therapy [41]. They have several advantages, such as their ability to reduce tension, decrease muscle activity, and prevent the harmful effects caused by bruxism and TMDs. Dylina defined occlusal splint therapy as “the art and science of establishing neuromuscular harmony in the masticatory system and creating a mechanical disadvantage for parafunctional forces with removable appliances” [43]. However, the use of occlusal splints prevents patients from achieving full intercuspation. As a result, patients need to position their jaws correctly, ensuring equal pressure on all teeth. This alignment helps the condyle to settle in a centric relation and encourages the development of new muscle and joint equilibrium [44].

Melo et al. evaluated the effectiveness of occlusal splint therapy, manual therapy, counseling, and a combination of occlusal splint and counseling in reducing pain in TMD patients. In the study, the combination treatment was observed to be more effective than the other treatments alone [22]. Rodrigues et al. compared the effectiveness of occlusal splint therapy with low-power laser auriculotherapy in patients with TMD, and the researchers examined both physical and emotional symptoms. Intriguingly, they discovered that both treatment methods yielded similar improvements in symptom relief [30].

Joint and Muscle Injections

Joint injections and muscle injections are two types of treatments used to address TMD symptoms. Pons et al. evaluated the viability of MR-guided navigation for administering botulinum toxin injections in TMD patients. In a prospective study, six patients underwent treatment with intramuscular botulinum toxin A injections. The therapy was found to be effective in 67% of the patients [31]. Vingender et al. assessed the clinical effects of platelet-rich fibrin, hyaluronic acid (HA), and platelet-rich plasma injections in the internal derangement of the TMJ. Their study concluded that there was no significant difference among the treated groups, suggesting that all injections were equally effective [29]. Sipahi Calis et al. investigated the effectiveness of botulinum toxin injections in the treatment of muscular TMD. Twenty-five patients received various treatments including drug therapy, drug combined with physical therapy, occlusal splint therapy, and botulinum toxin injections. The botulinum toxin treatment showed positive results in nine patients [27].

Arthrocentesis

Performed under local anesthesia, arthrocentesis is done to flush out the superior space of the TMJ. It aims to reduce intra-articular pressure and control pain. For joint space lavage, normal saline, steroids, botulinum toxin, HA, or anti-inflammatory agents are used. The procedure encompasses three key steps: separating the joint constituents, removing inflammation, and eliminating intra-articular effusion [41]. Arthrocentesis is effective for both internal derangement and inflammatory degenerative disorders of the TMJ, making it a recommended treatment modality. Furthermore, the procedure has demonstrated favorable outcomes in both short-term and long-term results, notably improving maximum mouth opening (MMO) and alleviating pain [45]. Polat and Yanik used the same arthrocentesis protocol to assess the efficacy of arthrocentesis therapy in 45 TMD patients. Arthrocentesis was found to be most effective in patients with disc displacement without reduction (DDWoR) [26].

Discussion

Even though clinical trials have identified methods with greater efficacy than others, the effectiveness of a particular treatment for TMDs varies between patients. This review describes the main findings from 20 selected studies, summarized in tables. The most relevant characteristics of these studies include the number of patients enrolled, baseline characteristics, age group and gender, type of TMD, type of treatment/therapy, frequency of application, duration of intervention, description of the procedure, outcome measures, and conclusions. The first study in this review was published in 1997, with most of the clinical studies published after 2015. While most of these studies were clinical trials, some were retrospective studies. The smallest trial conducted by Pons et al. enrolled a total of six patients, whereas the largest one by Gębska et al. (2023) enrolled 186 patients [17,31]. Half of the studies compared the effectiveness of a single treatment against a placebo group, whereas other studies evaluated multiple therapies and compared their effectiveness. The duration of the treatments varied from one week to six months. The efficacy of the treatments was assessed using various measures such as the VAS, active range of motion (AROM), MMO, and mandibular muscle mobility.

Although there are insufficient data to conclusively prioritize one treatment method over another for TMDs, some clinical trials have found that TENS [18] and low-level laser therapy [16] were not very effective in reducing pain or increasing MMO in patients. Several studies indicated that certain therapies did not show long-term results despite short-term improvement in symptoms [13-18,20-22, 24-27,30,31]. These therapies include low-level laser therapy, high-intensity laser therapy, laser acupuncture, photobiomodulation auriculotherapy, LIPUS, TENS, physical therapy, occlusal splint, manual therapy, counseling, and botulinum toxin A injections. However, four studies [19,23,28,29] reported treatments that demonstrated long-term effectiveness in reducing pain, improving MMO, and enhancing mandibular muscle mobility. These effective treatments include osteopathic manual therapy, remodeling dental anatomy, myorelaxation therapy, and intra-articular injections of HA, platelet-rich plasma, and injectable-platelet-rich fibrin.

Two studies focused on using botulinum toxin to treat TMD symptoms: Calis et al. reported a 67% efficacy [27], while the other documented a 36% effectiveness [28]. Additionally, two studies reported more than 90% improvement in TMD patients with combination therapies. One study found that a combination of manual therapy, occlusal splint, and counseling was highly effective in reducing pain (approximately 99%), depression (approximately 99%), and anxiety (approximately 99%) [22]. The other study employed a combination of surface electromyography and physical therapy, which was effective in reducing pain and improving mandibular muscle mobility and mouth opening in 99.9% of the patients [17].

Based on the outcomes reported in these studies, it is advisable to initiate treatment for TMDs after proper diagnosis via surface electromyography. Following diagnosis, a progressive approach should be adopted, starting with counseling, manual therapy, physical therapy, and splint therapy. If pain persists despite these initial treatments, the use of more invasive methods such as injections and arthrocentesis should be considered. Optimal results are often achieved by employing a combination of treatments. For instance, the combined use of manual therapy, occlusal splint, and counseling has produced the best results in reducing pain, depression, and anxiety. Similarly, a combination of surface electromyography and physical therapy has been effective in reducing pain, improving mandibular muscle mobility, and increasing mouth opening in 99.9% of the patients.

This systematic review stands out for its comprehensive approach to evaluating the efficacy of non-invasive and minimally invasive treatments for TMDs. The breadth of the literature search, encompassing a wide range of scientific databases up to December 2023, ensures an exhaustive inclusion of relevant studies, thereby enhancing the review's coverage and relevance. The analysis spans a diverse array of treatment modalities, ranging from physical and laser therapies to ultrasound, electrical stimulation, splint therapy, injections, and arthrocentesis, offering a nuanced perspective on the therapeutic landscape for TMD. A significant strength of this review is its dual approach to data synthesis, combining quantitative measures such as VAS, AROM, and MMO, with qualitative assessments of patient satisfaction and improvements in depression and anxiety scores. This methodology provides a comprehensive evaluation of treatment outcomes, considering both objective measures of improvement and subjective patient experiences.

Moreover, the review places a particular emphasis on the efficacy of combination therapies, highlighting the potential of integrated treatment approaches to yield superior outcomes. This focus aligns with a growing recognition in the medical community of the benefits of multidisciplinary care in managing complex conditions such as TMD. By prioritizing patient safety and cost-effectiveness, the review echoes the current healthcare imperative for value-based care, advocating for conservative, non-invasive treatments as the initial management strategy. This approach is not only aligned with best practice guidelines but also reflects a patient-centered perspective, emphasizing treatments that are both effective and minimally burdensome.

The timeliness of the review, incorporating studies up until December 2023, positions it as one of the most current analyses on the topic, making its findings highly relevant for both clinical practice and future research directions. The comprehensive and meticulous methodology, combined with a focus on patient-centered outcomes and the exploration of combination therapies, underscores the review's significant contribution to the body of knowledge on TMD treatment.

Despite the comprehensive approach and significant insights provided by this systematic review of non-invasive and minimally invasive treatments for TMDs, it is not without its limitations. One of the primary constraints stems from the inherent variability in study designs, treatment modalities, and outcome measures across the included studies. This heterogeneity complicates the direct comparison and aggregation of data, potentially affecting the uniformity of the conclusions drawn. Additionally, while the review encompasses a wide range of treatment approaches, the depth of analysis for each specific treatment could be influenced by the availability and quality of the studies. The majority of included studies focus on short to medium-term outcomes, leaving a gap in our understanding of the long-term efficacy and sustainability of these treatments for TMD.

Moreover, the patient populations in the analyzed studies were not always homogeneously defined in terms of TMD subtypes and severity, which could introduce variability in treatment response and efficacy. This variability underscores the challenge of generalizing findings across the broader TMD patient population. Another limitation lies in the potential publication bias, as studies with positive outcomes are more likely to be published than those with negative or inconclusive results. This bias could skew the overall assessment of treatment effectiveness presented in this review.

Furthermore, the review's focus on non-invasive and minimally invasive treatments, while valuable, means that comparisons with more invasive treatments were not systematically explored. This decision might limit the understanding of the full spectrum of therapeutic options available for TMD, particularly for complex or refractory cases where surgical interventions could be considered. Despite these limitations, this systematic review contributes valuable insights into the efficacy of various TMD treatments, providing a solid foundation for future research to build upon. Addressing these limitations through well-designed, long-term, multicenter studies with standardized outcome measures would significantly enhance our understanding of TMD treatment efficacy and patient care.

## Conclusions

Treatments for TMDs range from simple self-care practices and conservative treatments to injections and mildly invasive procedures. It is advisable to start with conservative, non-invasive, or mildly invasive therapies and reserve surgery as a last resort. Furthermore, combining different treatments often yields the best results. For example, it has been recommended to use surface electromyography in conjunction with physical therapy or a combination of manual therapy with an occlusal splint and counseling. In TMD patients, these specific combination therapies have led to improvements of over 90%, underscoring the effectiveness of a multi-modal treatment approach.
